# Vitamin D3 Promotes Structural and Functional Recovery After Vincristine-Induced Peripheral Neuropathy in Rats: An Experimental Study

**DOI:** 10.7759/cureus.34979

**Published:** 2023-02-14

**Authors:** Hüseyin Erdem, Levent Sarıkcıoğlu, Neslihan Boyan, Kamil Savaş, Nazmi Yaras, Ozkan Oguz

**Affiliations:** 1 Department of Anatomy, Cukurova University Faculty of Medicine, Adana, TUR; 2 Department of Anatomy, Akdeniz University Faculty of Medicine, Antalya, TUR; 3 Department of Biophysics, Kirklareli University Faculty of Medicine, Kirklareli, TUR; 4 Department of Biophysics, Akdeniz University Faculty of Medicine, Antalya, TUR

**Keywords:** chemotherapy-induced peripheral neuropathy, vincristine-induced peripheral neuropathy, vitamin d3, vincristine, peripheral neuropathy

## Abstract

Background

Vincristine-induced peripheral neuropathy (VIPN) is a distal axonopathy characterized by the loss of distal myelinated axons. This study aimed to assess the potential neuroregenerative roles of vitamin D3 using functional and electron microscopic analyses in a rat model of VIPN.

Methodology

A total of 40 female Wistar rats were randomly divided into four main groups: Group 1 (control, *n* = 10), Group 2 (vincristine, *n* = 10), Group 3 (vincristine + vitamin D3, *n* = 10), and Group 4 (vincristine + vehicle, *n* = 10). Vincristine was administered intraperitoneally at a dose of 0.15 mg/kg, for two weeks, to induce peripheral neuropathy. Following successful induction, vitamin D3 (500 IU/kg/day) and vehicle treatments were applied weekly over four weeks. Structural (electron microscopic analysis) and functional analysis (von Frey test, pinch test, and electrophysiological analysis) were performed to assess functional recovery after peripheral nerve impairment.

Results

Withdrawal responses to mechanical allodynia and pinch tests were significantly higher in the vitamin D3-treated group (*P *< 0.05). The electrophysiological analysis also supported these results. Electron microscopic evaluation revealed that the remyelinated nerve fibers in the vitamin D3-treated group (Group 3) had thick myelin sheaths and normal axonal morphology.

Conclusions

Our study demonstrated that vitamin D3 could promote functional and structural recovery in a rat model of VIPN. Further studies should be conducted to elucidate the underlying mechanisms by which vitamin D3 exerts its regenerative effects in VIPN, using alternative administration protocols.

## Introduction

Vincristine was introduced in the early 1960s as a chemotherapeutic agent. Since then, it has been effectively used to treat a wide range of types of both adult and pediatric neoplasms, including breast cancer, non-Hodgkin’s lymphomas, and leukemia [1]. Its antineoplastic effect is characterized by the disruption of microtubule dynamics. Specifically, vincristine prevents mitotic spindle assembling by inhibiting tubulin polymerization and microtubule incorporation, thereby delaying mitosis and consequently inducing apoptosis [2].

Chemotherapy-induced peripheral neuropathy (CIPN) is a common dramatic side effect of anticancer drugs. Vinca alkaloids are known to inhibit the polymerization of the β-tubulin subunit of microtubules, a key component in maintaining axonal continuity and promoting axonal transport [3]. Among the other vinca alkaloids, vincristine has more potent neurotoxic activity and almost all the vincristine-received patients are predictably affected by peripheral neuropathy (vincristine-induced peripheral neuropathy, or VIPN) [4]. It is a dose-dependent side effect, reducing the quality of life, and is frequently characterized by bilateral involvement of both sensory and motor nerves. Autonomic involvement is a rare and severe form of VIPN [5].

Numerous experimental studies and also clinical trials have been focused on the potential prophylactic and curative efficacy of alternative agents and protocols for the prevention of VIPN [6]. However, these methods are insufficient. Vitamin D3 (cholecalciferol) is an FDA-approved, fat-soluble secosteroid hormone that is mainly used for preventing rickets in practice. This hormone plays a variety of pleiotropic functions, particularly within the nervous system and immune system [7]. For instance, long-term low-serum vitamin D concentrations have been linked to lower brain volumes and (especially) larger lateral ventricles, as well as to a variety of neurodegenerative diseases, anxiety, depression, psychoses, and mental developmental defects [8]. Furthermore, Vitamin D3 has been reported to have regenerative roles on peripheral nerves after mechanical axonal injury models or ischemic insult [9-12].

Vitamin D receptors (VDRs) have an integral role in the organization of microtubules, which form the cytoskeleton. Calreticulin, a calcium-binding protein found in the lumen of the endoplasmic reticulum, initiates a series of reactions that enables the release of VDRs and their subsequent migration into the nucleus, after binding with Ca^2+^ in the cytoplasm [13]. A similar relationship between calreticulin and VDR was also observed when calcitriol was added to the fibroblast culture [14]. Furthermore, disruption of the tubulin structure through the drugs (nocodazole, colchicine, and vinblastine) has been shown to impede the aggregation and functions of the VDRs. Moreover, an increase in 24-hydroxy-cholecalciferol metabolites or a decrease in calcitriol synthesis has been demonstrated to cause depolymerization of microtubules [15].

Previous reports showed that vitamin D has several important activities in cytoskeleton formation and also has neuroregenerative and neuroprotective roles in peripheral nerves [9-12]. Therefore, in this study, we aimed to investigate the possible neuroregenerative roles of vitamin D3 after inducing peripheral neuropathy by vincristine administration in rats.

## Materials and methods

A total number of 40 female Wistar rats (200-250 g) were randomly divided into four groups: Group 1 (control, *n* = 10), Group 2 (vincristine, *n* = 10), Group 3 (vincristine + vitamin D3, *n* = 10), and Group 4 (vincristine + vehicle, *n* = 10). All animals were kept under standard laboratory conditions. Food and tap water were supplied ad libitum with an artificial light-dark cycle of 12 hours light on, 12 hours off. All experimental procedures were conducted per the Turkish Law on the Protection of Animals and were approved by the Local Animal Welfare Committee (Protocol no. B.30.2.AKD.0.05. 07.00/87).

Vincristine and vitamin D3 administration procedure and schedule

Vincristine was administered according to the following schedules: Monday through Friday, for two weeks, intraperitoneally (IP), at a dose of 0.15 mg/kg. At the end of the vincristine administration procedure, rats were evaluated by mechanical allodynia test and pinch test to ensure peripheral neuropathy induction was succeeded. After the observation of successful peripheral neuropathy symptoms, the vitamin D3 treatment procedure (and its corresponding vehicle treatment) was initiated.

Vitamin D3 (500 IU/kg/day) and vehicle administration procedure included four weeks and started two days after the successful peripheral neuropathy induction was observed. Vitamin D3 and vehicle treatments were applied weekly.

The study design was summarized in the Timeline document (Appendix).

Behavioral studies

Mechanical Allodynia

Rats were habituated separately in plexiglass cages with perforated bottoms (a mesh-like open grid of square holes ~5 mm × 5 mm), which allows access of the monofilaments to the hind paws. The test procedure for each rat began after 15 minutes of behavioral adaptation was provided or when exploring and major grooming activities ceased without a determined time restriction. The mid-plantar area of the left hind paw was determined for testing, avoiding the footpads, which are less sensitive to mechanical allodynia. von Frey hairs were touched perpendicularly to the mid-plantar surfaces until a little buckling was observed.

The von Frey test was employed to evaluate the efficacy of vincristine administration and monitor the recovery of peripheral neuropathy. The test involved stimulation of the hind limb with increasing stiffness of von Frey hairs and gauging the response. A sharp withdrawal or flinching of the stimulated hind limb was taken as a positive response, while ambulation or uncertain responses were considered negative. To ensure accuracy, a cutoff value of 15.10 g was established to prevent the whole limb raising instead of the desired withdrawal reflex.

Pinch Test

The sensory functional recovery was evaluated by the pinch test. The sole of the left hind limb was gently pinched using forceps (3 cm long), as previously described [16]. A gradual scale with four levels was used to assess the withdrawal reflex (sensory recovery). The grades were no response (Grade 0), mild response (Grade 1), moderate response (Grade 2), and full response (Grade 3). Rats responding with full withdrawal reflex to pinch stimulation were recorded as Grade 3.

Electrophysiological Analysis

At the end of the fourth postinduction week, rats were anesthetized with an intraperitoneal injection of a cocktail of xylazine HCl (15 mg/kg) and ketamine (100 mg/kg) mixture and then placed in the prone position and warmed using a heating pad (Small Animal Temperature Control Unit, D79232, Hugo Sachs Inc., March, Germany). Electrical stimulation was performed using coated steel needle electrodes and a biphasic pulse generator (STIMOLA, BIOPAC MP150 Systems Inc., Santa Barbara, CA, USA), previously calibrated for the study. The electrodes were located 1 cm proximal to the first branch point of the left sciatic nerve. Supramaximal square pulse (0.1 ms duration and 1 Hz) was applied for sciatic nerve stimulation. Paired electromyography (EMG) electrodes were inserted transcutaneously along the length of the medial gastrocnemius, lateral gastrocnemius, and soleus muscles. EMG signals were amplified (1,000-5,000 Hz) and band-pass filtered (10-5,000 Hz) using a differential amplifier (BIOPACEMG100C, BIOPAC MP150 Systems Inc., Santa Barbara, CA), before being digitized and recorded (BIOPAC MP150 Systems Inc.). An average of 20-30 responses were used to calculate the compound muscle action potential (CMAP).

Electron microscopic evaluation

After completion of the electrophysiological analysis, rats were sacrificed by high-dose ether anesthesia. After transcardial fixation of all rats, 1-1.5 cm left sciatic nerve segments were harvested and fixed with 4% glutaraldehyde in 0.1 M Sorensen’s phosphate buffer solution (pH = 7.3). Then, it was postfixed with 2% osmium tetroxide in the same buffered solution after dehydration through a graded series of ethanol. Samples were embedded in epoxy resin (Araldite CY212, Agar Scientific Ltd, Stansted, UK). Semithin sections were stained with toluidine blue and were examined with a light microscope (Olympus CX41, Olympus, Tokyo, Japan). Afterward, ultrathin sections (40-60 nm) were contrasted with uranyl acetate and lead citrate and finally examined with a Zeiss LEO 906E electron microscope (Zeiss, Oberkochen, Germany).

Data analysis

All measurements were carried out blindly. Normally distributed data were analyzed by one-way analysis of variance (then Tukey's test as a posthoc test), and the values that did not fit the normal distribution were analyzed by Kruskal-Wallis. Pinch test data were analyzed by chi-square test. Kruskal-Wallis test and Dunn test were used to evaluate the electrophysiological and mechanical allodynia test data. SPSS Version 20.0 (IBM Corp., Armonk, NY, USA) package program was used in all statistical analyses. A *P*-value of less than 0.05 was considered statistically significant.

## Results

Behavioral studies

Mechanical Allodynia

**Figure 1:**
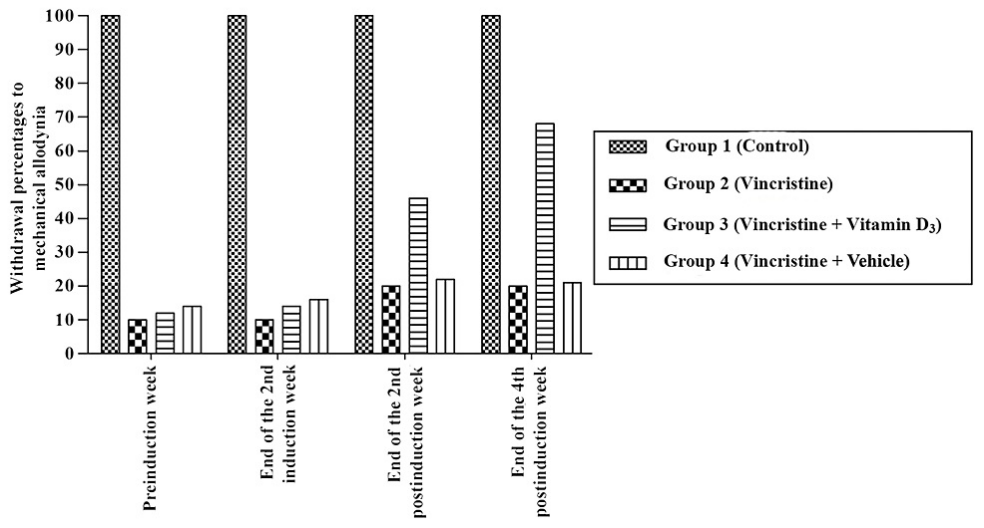
von Frey test. Mechanical allodynia percentages at the fourth postinduction week.

At the end of the 4th postinduction week, the withdrawal reflex of the vitamin D3 treated group (Group 3) was significantly higher than those of Group 2 and Group 4 (*P *< 0.05). The same pattern was also observed in the second postinduction week (*P *< 0.05) (Figure 1).

Pinch Test

**Figure 2:**
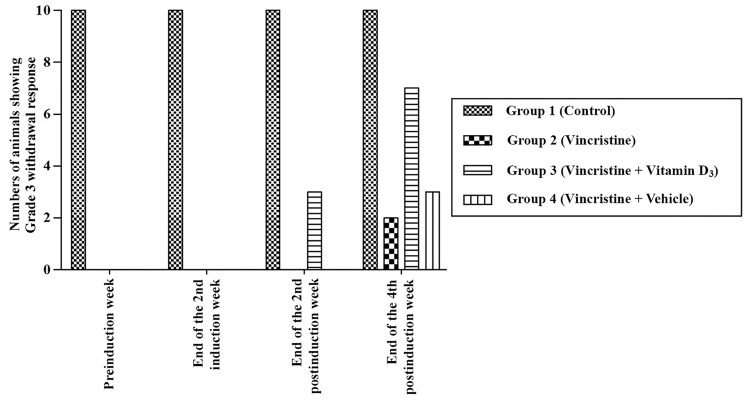
Pinch test. Pinch test results were noted at the preinduction week, at the end of the second induction week, and at the end of the second and fourth postinduction weeks. Analysis of the data revealed a significant difference (*P *< 0.05) between Group 3 (vitamin D3-treated group) and Group 4 (vehicle-treated group), at the end of the fourth postinduction week.

At the second and fourth postinduction weeks, Grade 3 withdrawal reflex (full withdrawal reflex) to pinch stimulation was observed to emerge in the vitamin D3-treated (Group 3) and vehicle-treated (Group 4) groups, respectively. In the second postinduction week, only the vitamin D3-treated animals (Group 3) showed full withdrawal reflex. At the fourth postinduction week, seven animals of the vitamin D3-treated group (Group 3) exhibited full withdrawal reflex. Furthermore, a statistically significant difference (*P *< 0.05) was found between the vitamin D3-treated (Group 3) and vehicle-treated (Group 4) groups in terms of the overall levels of withdrawal reflexes (Figure 2).

Electrophysiological Analysis

**Figure 3:**
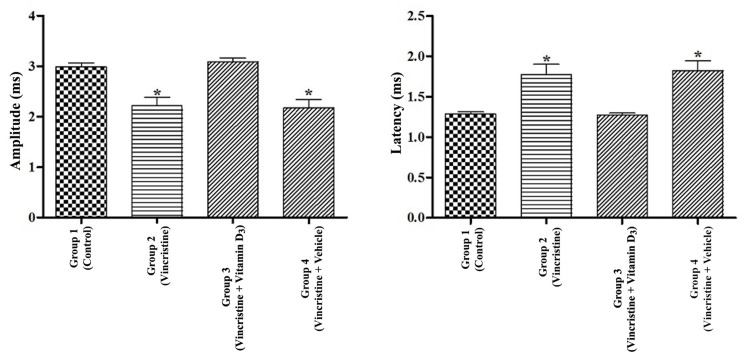
Electrophysiological analysis. Peak-to-peak amplitudes and latency of the sciatic nerve in all groups. Data were analyzed by ANOVA (posthoc Tukey's test). Peak-to-peak amplitude was significantly increased and the latency was significantly decreased after vitamin D3 administration in comparison to those of Group 2 and Group 4. *Significant difference (*P* < 0.05) versus vitamin D3-treated group (Group 3). ANOVA, analysis of variance

The latency and CMAP amplitude levels were significantly different between the control (Group 1) and vincristine-treated (Group 2) groups (*P *< 0.05). Vitamin D3 treatment significantly decreased latency and increased CMAP amplitude compared to both vincristine treatment (Group 2) and vehicle treatment (Group 4; *P *< 0.05). Moreover, there was no statistical significance observed between the two groups (Group 2 and Group 4) in terms of CMAP amplitude and latency (Figure 3).

Electron Microscopic Evaluations

**Figure 4:**
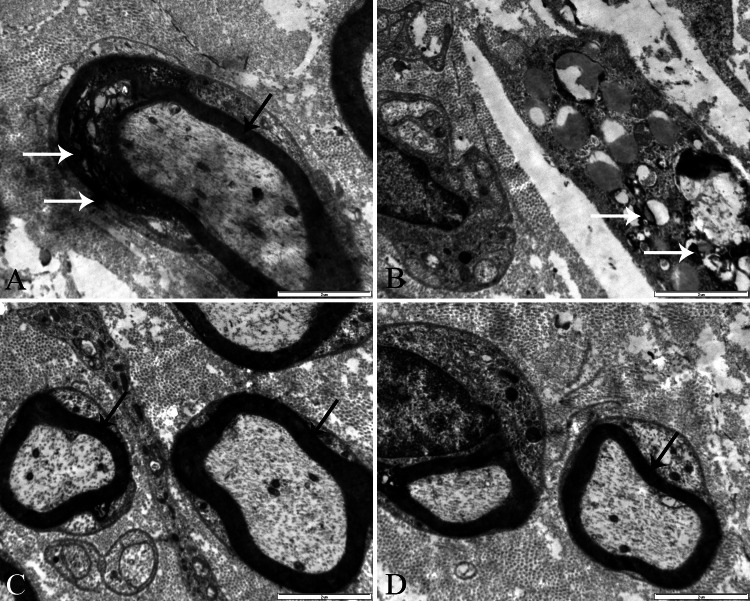
Electron microscopic analysis. Electron microscopic appearance of the sciatic nerve samples in Group 2 (vincristine), Group 3 (vitamin D3-treated group), and Group 4 (vehicle-treated group). The white arrowhead shows the myelin residues, and the black arrowhead shows the remyelinated nerve fibers. (A) Degeneration and disorganization in myelin fibers were observed in myelin sheath layers in sciatic nerve samples of Group 4. (B) Myelin debridements in sciatic nerve samples of Group 2. (C and D) Axonal structures and remyelinated nerve fibers in Group 3.  Relatively intact axons and myelin sheaths were striking compared to other groups.

Electron microscopic evaluation of the control group (Group 1) indicated the normal sciatic nerve morphology containing typical nerve fibers without myelin deformation. On the other hand, degenerated nerve fibers characterized by myelin debridements and separation between myelin compartments were observed in samples of the vincristine-treated group (Group 2). Additionally, quite thin remyelinated nerve fibers were noticed in the same samples (Group 2). The appearance of the nerve samples belonging to the vitamin D3-treated group (Group 3) was striking: remyelinated nerve fibers had thick myelin sheaths and also axonal structures had a normal appearance. Numerous phagocytic cells (macrophage or Schwann cells) with myelin residues were observed in the samples of the vehicle-treated group (Group 4; Figure 4).

## Discussion

In this study, intraperitoneal vincristine administration for two consecutive weeks led to the significant development of mechanical allodynia. Behavioral changes were also consistent with the previous reports that document the development of neuropathy symptoms, which were induced by vincristine administrations.

In this study, the efficacy of sensory functional recovery was assessed using a pinch test. Nakamura et al. investigated the dose-dependent alterations of VIPN after crush injury of the sciatic nerve in a mice model [17]. They evaluated pain sensation with a modified type of pinch test which was characterized by pinching the third and fourth toes of the left hindlimb with forceps [17]. Results revealed that compared to the 0.05 and 0.25 mg/kg vincristine-treated mice groups, recovery of pain sensation was significantly delayed in the 1.25 mg/kg vincristine-treated mice group, at approximately 26 days. In our study, only three rats showed full withdrawal reflex (Grade 3) in the fourth postinduction week in the vincristine-treated group (Group 2). However, in the vitamin D3-treated group (Group 3), full withdrawal reflex (Grade 3) started in three rats in the second postinduction week and seven rats showed full withdrawal reflex (Grade 3) in the fourth postinduction week. In a comparison of Nakamura et al.’s and our results, there was a striking difference in terms of sensorial recovery time [17]. This difference could be attributed to the use of different species of experimental animals, as well as the difference in doses and administration schedules.

Authier et al. studied behavioral, electrophysiological, and histological changes after the dose-dependent vincristine administration [18]. Their results indicated that the 150 µg/kg dose treatment (equivalent to 0.15 mg/kg) significantly decreased the withdrawal threshold and peripheral nerve conduction velocity. They also reported that axonal swelling and disordered axons with undamaged myelin sheaths were observed in sciatic nerve samples [18]. These findings were consistent with our observations. However, in our study, myelin sheaths of the vincristine-treated group (Group 2) were thinner than those of the control (Group 1) and vitamin D3-treated groups (Group 3).

The rat models of VIPN vary, but similar doses of daily administrations (Monday through Friday, for two weeks), which aims to simulate the clinical practice were commonly accepted [19]. Multiple routes of administration have been reported in the literature, including intraperitoneal injection, which is preferred over intravenous injection due to the risk of phlebitis associated with repetitive IV applications [20]. To effectively generate neuropathy symptoms, doses of 0.05 and 0.15 mg/kg have been used in certain laboratories; however, it has been suggested that the 0.15 mg/kg dose is more efficacious, as evidenced by functional and morphological findings [21].

Our literature search revealed that the regenerative role of vitamin D in the VIPN model has not been investigated. However, the application of either vitamin D2 or vitamin D3 at higher (500 IU/kg/day) and lower (100 IU/kg/day) doses were found to have neuroprotective or neuroregenerative effects on experimental peripheral nerve injury models [10-12]. It was observed that vitamin D3 was more effective, delivering considerable electrophysiological and locomotor recovery when administered at higher doses [12].

Lower serum vitamin D levels were related to diabetic peripheral neuropathy in geriatric diabetic patients. Anju et al. demonstrated an alternative technique to treat peripheral neuropathic pain in type 2 diabetic patients with low-level laser therapy [22]. They suggested that the alleviation of symptoms associated with neuropathic pain could be attributed to the increase in serum magnesium and vitamin D concentrations [22]. Grim et al. conducted a prospective trial to examine the nutritional risk factors involved in the development of CIPN and found a significant difference in serum vitamin D levels between patients without CIPN symptoms (38.2 nmol/L) and those with CIPN symptoms (25.6 nmol/L) [23]. However, this study focused on the regenerative roles of vitamin D after the induction of VIPN. Although serum vitamin D levels were not monitored, the results of this study suggest that vitamin D treatment may be therapeutically beneficial for VIPN, in addition to its protective effects.

Vitamin D has been demonstrated to play crucial roles in axogenesis, myelination, and axonal transportation in peripheral nerves [9-12]. Cornet et al. showed that the presence of calcitriol in rat Schwann cell culture increased the expression of VDR and nerve growth factor genes, suggesting that vitamin D may actively involve in the regeneration processes of the peripheral nerves [24]. Chabas et al. reported that vitamin D2 has significantly enhanced axogenesis and axon diameter, as well as improved the responses of sensory neurons [11]. They also demonstrated the high dose of vitamin D3 treatment (500 IU/kg/day) has significantly induced the electrophysiological and locomotor recovery while increasing the number of newly formed or preserved axons in the proximal end, the mean diameters of axons in the distal end, and neurite myelination in both ends [12]. Moreover, Montava et al. investigated the therapeutic benefit of vitamin D supplementation after a facial paralysis model in rabbits and found that vitamin D3 treatment significantly increased functional recovery and myelination [10]. Similarly, our previous study demonstrated that administering vitamin D3 at a dose of 500 IU/kg/day to rats in an ischemic peripheral nerve injury model, for four weeks, resulted in neuroregenerative indicators that reached control-like values [9].

In our study, electron microscopic evaluations revealed that distal myelinated axons were affected in the vincristine-treated group (Group 2), a phenomenon that is consistent with the description of VIPN as a distal axonopathy [25]. Furthermore, Chabas et al. reported that the addition of calcitriol to Schwann cell or DRG cultures resulted in the alteration of 40 genes, several of which (e.g., Igf1, Metrn, Limk1, Ulk2, Prx, Tspan-2, Spp1) are known to play essential roles in axogenesis and myelination. Besides its several functions, Spp1 (osteopontin) is a well-known vitamin D-regulated cytokine that has been associated with myelination and axogenesis [12]. While transcriptomic evaluation was not utilized in this study, the available evidence surrounding osteopontin and the findings of Chabas et al., may support the hypothesis that vitamin D exerts a neuroregenerative effect in the VIPN model [12].

There is another major component of VIPN formation, oxidative stress. Vincristine administrations have been reported to cause depletion of superoxide dismutase (SOD), catalase (CAT), and glutathione peroxidase (GPx) activities. In addition, the levels of the final toxic product of lipid peroxidation, the malondialdehyde (MDA, an indicator of neuronal death and cytotoxicity), have been increased after the vincristine administration [26]. Furthermore, oxidative stress causes vascular deformation, leading to hypoxic endoneurial stress and finally leading to reduced nerve conduction velocity. Our previous study demonstrated that the administration of vitamin D after ischemia-induced injury to the peripheral nerves elicited regenerative changes in markers of oxidative stress [9]. In this study, vitamin D treatment significantly reduced the latency and increased the CMAP amplitude. These results, which we revealed in the electrophysiological analysis, may support the antioxidant effect of vitamin D.

Limitations

This study was limited in its scope by the lack of certain analysis techniques. First, serum vitamin D3 levels were not monitored, which could have provided further insights into the role of vitamin D3 in the neuroregeneration process. Second, transcriptomic analyses were not performed to assess the expression of genes associated with vitamin D metabolism. Third, oxidative stress indicators were not analyzed, which could have explained the antioxidant potential of vitamin D3 in the VIPN model.

## Conclusions

To the best of our knowledge, this is the first study investigating the neuroregenerative effect of vitamin D3 in an animal model of VIPN. Behavioral and morphological evaluations of this study revealed that vitamin D3 administration is capable of effectively ameliorating the peripheral neuropathy caused by vincristine. Further investigation into the genomic functions and physiological pathways underlying this phenomenon is necessary to determine the potential mechanisms by which vitamin D3 induces neuroregeneration in CIPN or VIPN. We also suggest that vitamin D3 should be administered in different schedules (before, during, and after the chemotherapy period) and be compared to evaluate their effectiveness with each other.

## Figures and Tables

**Figure 5:**
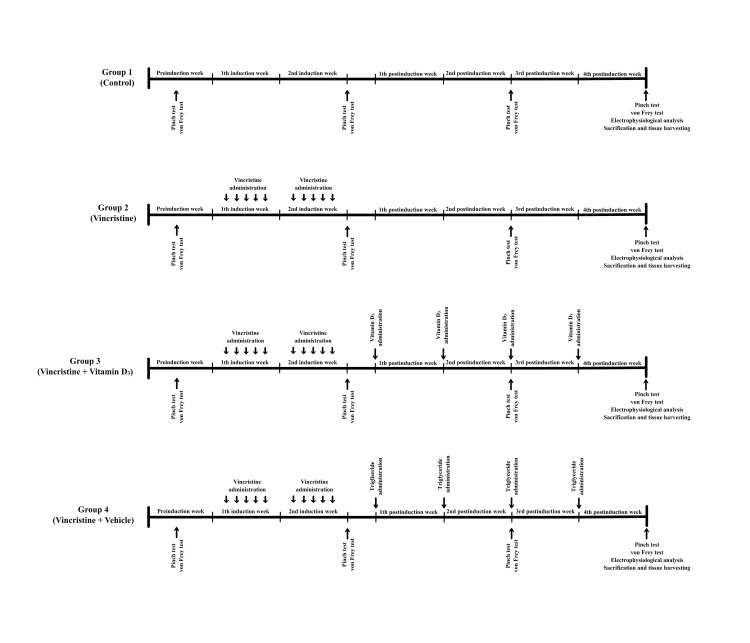
Timeline. The study design was summarized in the Timeline document.
